# Volunteer Services in Palliative Care by Third Age University Students

**DOI:** 10.3390/healthcare12242591

**Published:** 2024-12-23

**Authors:** Gönül Düzgün, Yasemin Kılıç Öztürk, Gökşen Polat, Sevnaz Şahin

**Affiliations:** 1 Vocational School First Aid and Emergency Program, Izmir Tınaztepe University, Buca, 35400 Izmir, Türkiye; goksen.polat@tinaztepe.edu.tr; 2 Izmir Medical Faculty，University of Health Sciences, Tepecik Training and Research Hospital, 35020 Izmir, Türkiye; yasemin.ozturk2@saglik.gov.tr; 3 Department of Internal Medicine, Division of Geriatrics, Ege University Hospital, 35100 Izmir, Türkiye; drsevnaz@gmail.com

**Keywords:** palliative care, active aging, volunteer training, qualitative research

## Abstract

Background: Volunteering is a type of support that provides high motivation and supports social participation during the active aging process without any financial reward. Volunteering services provided by an active older person not only provide free services to the community where needed but also help individuals feel valued by creating a social environment, thereby increasing their resilience. The aim of this study was to determine the views on volunteering in palliative care services among older individuals over the age of sixty who attend the Ege University of the Third Age [U3A] and outline the volunteer profile of older students after receiving palliative care training. Methods: This study was conducted using a qualitative research method. The study population consisted of seventy students from the Ege U3A in İzmir who met the inclusion criteria. Before the training, the U3A students were given a brief pre-test about palliative care and volunteering, followed by 2-day, 16 h basic palliative care training. After the training, the students were asked to respond to written questions about volunteering in palliative care, and their responses were collected in their own handwriting. For the data analysis, a thematic content analysis was conducted using MAXQDA 20, a qualitative data analysis program. Results: In this study, the average age of the 70 University of the Third Age students were 67.47 (60–89) years. The participants were 75% women; 85% were married, 40% lived with their spouse, and 37.14% had been students at the University of the Third Age for 3 years. After the thematic analysis, six main themes emerged: physical and social support, educational support, medical/clinical support, financial support, spiritual support, and caregiving for pediatric patients. Conclusions: In our study, it was clearly observed that older students enrolled in the Ege U3A, who had a high potential for volunteering, were willing to work voluntarily in palliative care within the limits of their physical abilities and resources. This research, which aimed to create a volunteer profile in palliative care, allowed older students to find suitable roles for themselves and increased their motivation to provide this unpaid service. Based on this, it aimed to establish an effective start and an encouraging practice for the development of a pilot study, which is needed for Türkiye.

## 1. Introduction

“Before meeting the patient, I learn about their preferences and needs to make appropriate preparations. If permitted, I can dress them in clothes that suit their taste and set aside some for them to wear once they have recovered, hoping to make them happy and give them hope by telling them they will be able to wear these clothes later. I find out their favorite music in advance and record it on my phone; if they wish, I can listen to the recorded music with them and sing along. I learn about books they like and, if they want, I can read them, then leave the books for their own reading later. I listen to their stories and try to engage in conversation. If there is a family photo album, I would obtain it from their relatives, look through it with them, and listen to their memories if they wish to share. At the end of the day, I review the time we spent together and make sure to convey my intention to return. The hope of spending another day together in a better and healthier way will be beneficial for both them and me.”

The statements above are expressions from a volunteer who participated in our study, written exactly as they were originally provided. We believe that the time has come to transform this potential that older individuals have for healthy aging into a real dynamic, especially for a developing country such as Türkiye in the field of palliative care.

Volunteer work can best be defined as a free, non-profit activity that usually serves the common good. Working as a volunteer supports a person’s positive attitude towards life. Helping someone else for free at any age provides a high level of motivation. Conducting volunteer work feels like therapy to a person and provides motivation to engage in it constantly [1]. The World Health Organization [WHO] classifies chronological ages as follows: older adults between the ages of 65 and 74 years as the youngest-old, those between the ages of 75 and 84 years as the middle-old, and those aged over 85 years as the oldest-old [2]. Being able to live actively during old age is an important criterion for successful aging [3]. Active aging is a multi-dimensional concept influenced by several factors, such as lifelong learning, physical health and functionality, lifestyle, environmental conditions, and social inclusion.

The most productive way of active aging for the elderly is to participate in volunteer work. The volunteer work of this population group can be understood as a valuable resource, as older people have freer time availability and can contribute a wealth of experience. Furthermore, volunteering is seen as an opportunity for older people, as it offers opportunities to participate in social life, experience social inclusion as well as recognition, and able to engage in meaningful activities. Therefore, it is not surprising that in Austria too, the highest volunteering participation rate of 57% occurred for the 60–69 age group and that 43% of 70–79-year-olds still volunteer [4]. Furthermore, the engagement of older people benefits not only volunteer organizations and society but also the individuals themselves. Many studies have shown that volunteering by older people is associated with better physical and mental health [5], a lower probability of illness, better mental well-being, greater life satisfaction, lower mortality, and better general well-being. This list includes aspects that people want for old age or need for successful aging [1].

Lifelong learning plays a key role in older individuals expressing themselves, participating in community life and employment, and developing skills and experience, and it also strengthens intergenerational communication and experience transfer. Studies indicate that there is a linear relationship between active aging and lifelong learning. U3A is one of the best examples of these studies [6]. U3A opened its doors to individuals over the age of 60 in 2016 as part of a lifelong learning initiative under a social responsibility project at Ege University. The Ege U3A, coordinated by the Department of Geriatrics at Ege University and involving volunteer academics from various faculties of the university, is a social responsibility project that includes both theoretical and practical courses. The theoretical courses cover a wide range of topics, from health preservation and vaccination to cosmology and history. Some of the practical courses include folk dance, psychodrama, choir, and knitting. The duration of the program is 3 years. U3A not only strengthens individuals physiologically, psychologically, and socially but it also contributes to the development of new roles [7]. These universities first emerged in France in 1973 [6,8]. Since the training programs are shaped according to regional needs, there is no standard curriculum. When examining examples in our country, no palliative care training specifically aimed at the older population has been found. It is important for older individuals to understand the concept, scope, and purpose of palliative care.

Particularly, in the aging global population, deaths caused by infections have been replaced by deaths due to chronic diseases. Consequently, aging individuals appear to be at a higher risk for chronic illnesses, and their need for symptomatic care related to these diseases has become more prominent. Understanding the content of this care forms a preliminary preparation phase for evaluating how well the provided services will meet expectations and for identifying the required areas of care. In particular, collaborating with healthcare professionals will provide an advantage in meaningfully maintaining social support for patients [9].

One of the best examples of creating a strong social support network through individuals who volunteer to join a team in palliative care in Germany. In Germany, individuals who volunteer to join a team in palliative care are representatives of normal life within the interdisciplinary team. They support the care team, establish connections between the institution and daily life, bring various skills and experiences to the team, and take on tasks such as conversing with patients or their families, reading books, going for walks, accompanying patients at night, and indirectly participating in bureaucratic tasks and other activities [10,11]. U3A students receive services from volunteer academics and observe what it means to serve in a voluntary job. Therefore, they are a very suitable group for volunteering, as they provide social and psychological motivation. The desire of U3A students to engage in active aging, their free time, and their high instinct to be useful to society after receiving education increase their volunteering potential.

In Türkiye, involving older individuals as volunteers in palliative care is highly valuable for promoting positive outcomes, such as continuing to produce based on healthy aging, strengthening social connections, and providing motivation through voluntary work. It allows older individuals to find and maintain a meaningful place in life to the extent that their competencies permit at every age. The aim of our research was to identify the views of U3A students who aim for active aging regarding volunteering in palliative care after receiving training on the subject and to outline the profile of older individuals in Türkiye who are interested in volunteering in palliative care.

## 2. Methods

This study used a qualitative research method. The study population consisted of Ege U3A students. A total of 70 students from Ege U3A, who were enrolled and had received 16 h of theoretical training in palliative care, were included in the study. The theoretical training program included the following topics: “Definition of palliative care (what is palliative care and what is it not?), palliative care practices in Türkiye, palliative care practices in the world, symptom management in palliative care, making a medical will, end-of-life care and communication with the dying patient, the health of caregivers in palliative care, and volunteer work in palliative care”.

The following inclusion criteria were used for this study:

- Being a U3A student;
- Being 60 years of age or older;
- Having completed 16 h of training in palliative care;
- Having no physical or sensory disabilities [e.g., writing, hearing/speaking difficulties];
- Being found healthy after a comprehensive geriatric assessment;
- Being willing to participate in the study.

### 2.1. Data Collection Method

This study was conducted between November 2023 and May 2024 with individuals over the age of sixty at Ege U3A in İzmir. All the students who met the research criteria were included in the sample. The data for the study were collected using an individual information form and a semi-structured interview form. During the pre-test phase, the individual information form and close-ended questions regarding the concept of palliative care and their past volunteer experience were administered. Subsequently, 2-day (totaling 16 h) palliative care training was provided.

After the training, a consent form was signed, and the purpose of the study was explained. The students who agreed to participate underwent a comprehensive geriatric assessment, and those without health problems affecting their ability to listen to and understand the lessons were included in the study. Socio-demographic information was collected using the individual information form. In the open-ended interview form, the participants were asked to write in their own handwriting and of their own free will whether they would like to offer support in the field of palliative care and the areas in which they would be willing to volunteer. It was explained that there would be no sanctions, obligations, or requirements regarding what they wrote, and if they did not wish to volunteer, they were asked to clearly express that in writing as well.

### 2.2. Data Collection Tools

#### 2.2.1. Individual Information Form

The participants were asked about their age, gender, marital status, family structure, how many years they have been a U3A student, what the concept of palliative care means to them, whether they had received palliative care training before, and their experience or intention to work as a volunteer.

#### 2.2.2. Open-Ended Interview Form

The interview form included 1 open-ended question and 2 yes/no questions aimed at revealing the volunteering profiles of individuals. The questions from the interview form are provided below.

1. After receiving training in palliative care, would you consider working as a volunteer to support palliative care? Yes/No
2. If your answer to the above question is ‘yes,’ in which areas and what kind of support would you like to provide as a volunteer for patients after receiving training in palliative care?
3. Would you like to be part of the volunteer pool for supporting palliative care patients and stay in communication with the training team? Yes/No

### 2.3. Data Analysis

In the data analysis, a qualitative content analysis was conducted. After transcribing the open-ended responses, a thematic content analysis was performed using MAXQDA 2020.1, a qualitative data analysis program. The researchers identified the themes, participants [volunteers were coded as V1, V2, V3], and sub-themes that represented the codes through iterative discussions until a consensus was reached [12].

#### Ethical Considerations

Permission was obtained from the Medical Research Ethics Committee of Ege University, decision No. 23-9T/58, on 7 September 2023. The Ege U3A students who agreed to participate were informed about the study and their written consent was obtained. The participants were also informed that they could withdraw from the study at any stage.

## 3. Findings

The average age of the 70 students included in the study was 67.47 (60–89) years. A total of 85% of the participants were married, 40% lived with their spouse, and 37.14% had been students at the Ege U3A for 3 years. It was noted that none of the students knew the meaning of palliative care before their training, and none had previously received palliative care training. Prior to the training, it was found that 82.85% of the students had not previously provided volunteer services, and 72% had not considered working as a volunteer.

After the training, 90% of the students expressed a desire to work as volunteers in palliative care, and 87% indicated that they wished to stay in communication within the volunteer pool. The areas in which the older individuals indicated they could or would like to provide support, based on their written statements, were categorized into five dimensions. Three experts in the research evaluated the written statements separately and extracted themes. Then, the experts reviewed the themes created together, and as a result, six main themes were reached without any sub-theme groups. These were as follows: physical and social support, educational support, medical/clinical support, financial support, spiritual support, and caregiving for pediatric patients.

### 3.1. Social and Physical Support Dimension

The day should begin with a sweet smile, followed by approaching the patient with that warmth and assisting with their daily self-care. I help them with physical activities as desired [V1, V11, V18].

I would like to provide physical care if health professionals teach me how to do it [V18].

I can assist with personal care and help with exercise [V23], [V36], [V53].

I would take on patient care for specific time intervals, such as 1–2 h a day or one day a week [V45].

I can assist with the patient’s nutrition and personal care [V46], [V47].

I would cut their hair and nails and ensure body hygiene [V55].

Then, I try to uplift their energy by engaging in conversations they enjoy, providing an environment that will be beneficial to them as much as possible. I make sure they feel and live in that room as if it were their home [V1].

I would play games, read books, take them outside depending on their condition, and listen to their memories [V3].

I would read books to patients for an hour or listen to them [I observed that older individuals have a great need to share their life experiences, and conversing always relaxes and calms them] [V4].

I would like to bring a meal to a palliative care patient and give their family member a day of rest, even if just for one day [V5].

I would try to distract my hospitalized friend or someone in need by discussing comforting topics [telling jokes, making humorous remarks] to help them shift their thoughts, even if just a little [V7].

At suitable times, I would try to help others in need by engaging in conversations, reading books, or doing their shopping [V10].

I would be present for tests and treatments to support the person and ensure their feelings are positive. If they are anxious or scared, I would find a topic or activity to distract them [e.g., reading a book] [V11, V31, V32, V33].

For a person who has lost their sight, you could describe the facial expressions and environment while watching a movie. I would like to be someone’s eyes, ears, or hands and feet [V12].

I can assist with their shopping and any needs outside the hospital [V13].

I would like to read books and poems, find topics of interest to them to discuss, and help open up their inner world to relax them [V14], [V40].

I would like to solve puzzles, play chess, and teach Alzheimer’s and dementia patients, sharing my knowledge to be helpful [V18].

Washing their clothes, boosting their spirituality and motivation, and providing psychological support are also very valuable [V19].

I would like to share that I can easily provide support on specific days and times for tasks related to the outside world that those in need of palliative care [in a home setting] might have difficulty managing, such as paying electricity and water bills, handling applications to official authorities, and other bank or tax office transactions that they cannot attend to themselves [V21].

I would communicate with the family and provide support to them. I would also interact with the patient. I can arrange appointments for the patients and take them to check-ups. I would facilitate visits with people the patient wants to see. Additionally, I can help with household chores [V23].

I can support individuals by staying with the patient for a couple of hours if they need to go to a health center or hospital to obtain supplies or see a doctor and cannot leave the house. If the patient and their family can go outside, I can take them for a drive to get fresh air and help them get away from their current environment [V24].

I can feed the patient, administer their medication, and measure their blood pressure. If the patient can speak, I can chat with them; if they enjoy reading, I can read books to them [V25].

I would like to contribute to the process by giving presentations on music and poetry for palliative patients [V26].

I would try to help older patients with walking, participating in their shared memories, and assisting with tasks like shopping and banking [V28].

Since I attend folk dance classes, I would also provide support in this area [V29].

I can help a conscious patient relax by deepening the conversation to express their current thoughts in words, feed them, and manage their medication [V30].

I can care for patients without family. I would sit by their side and listen to them. I would talk to them to make them feel that they are not alone and that there are people who care about them. I can provide emotional support, boost their spirits, and follow up on hospital procedures [V37].

As a man, although I may not be able to provide as much service as women, I would like to assist with technical matters. I would like to work on repairing and maintaining equipment such as chairs, beds, and other tools used by patients and staff [V44].

Physical health is just as important as mental and emotional well-being. I practice a sport that has a remarkable effect on individuals by integrating both. I can do simple Tai Chi with them, and face yoga is also included [V48].

Caregiver Burden I know that my late spouse, A, experienced a lot of difficulties due to dementia. For this reason, I want to help caregivers in the hospital [V2].

I would provide support to caregivers according to their needs [V3], [V35].

I had my parents and three siblings, but I felt alone and abandoned. I deeply experienced loneliness, helplessness, and social isolation. I would like to share and alleviate loneliness by talking with someone in an analogous situation [V6].

If possible, I would support caregivers by completely relieving them for a while so they can rest, relax, and clear their minds and spirits. I would ensure that their loved ones are fully cared for while the caregivers are away for a few hours or a few days, allowing them to rest and recuperate [V9].

I would assist with tasks such as cleaning and laundry for the caregivers of patients who are bedridden for long periods [V13].

During the caregiver’s rest periods, I could accompany the patient myself [V15].

Volunteers’ involvement with patients relieves the caregivers. Being away from the environment and taking a breather not only boosts their spirits but also provides hope for the future [V17].

I can provide psychological support to the patient’s caregiver and assist them on certain nights. For example, I would ensure they get some rest and offer help through communication, as far as my abilities allow [V20].

I can visit the caregivers and listen to their concerns while chatting with them over sweet and salty homemade snacks [V22].

I can stay by the patient’s side to give the caregiver a break, handle external errands such as pharmacy, market, and grocery shopping, and cook meals according to their preferences [V25].

I can give the caregivers a rest for 3–4 h and visit them [V27, V33, V38, V41, V34].

I can give him/her a break to rest his/her companion, allowing him/her to get away from the hospital environment and take a day for himself/herself by walking and getting fresh air [V30].

### 3.2. Educational Support Dimension

In the social volunteer project, I can support by taking courses or seminars for training if needed [V8].

I would like to work in education and consultancy services within palliative care [V42].

I can volunteer to provide information and suggestions for Alzheimer’s patients [V43].

If I were to volunteer, I would encourage the widespread adoption of volunteering by motivating young students to participate in such activities, even if only for a short period [V55].

I could organize small-scale celebrations for patients on special occasions, as long as their health permits [V56].

When it comes to education and training, I would volunteer to work in every aspect. I would read and explain and use my training in drama and theater to dramatize various topics [V57].

### 3.3. Medical/Clinical Support Dimension

I would like to work as a physiotherapist in palliative care as a volunteer. Palliative care patients may need assistance with certain movements they are unable to perform [V67].

After retiring, I participated in various courses such as first aid and I want to contribute as well [V29].

I can measure the patient’s blood pressure/temperature and provide massages [V39].

If the patient is bedridden and unable to turn themselves, I can apply medication recommended by the doctor to prevent bedsores [V41].

I think I can support all these tasks, such as maintaining proper nutrition, personal care, administering medications on time, and providing ongoing medical support at home or from healthcare institutions [V50].

I can share my knowledge and experiences with dialysis patients and their families. I can provide training on how to change fluids during peritoneal dialysis, apply hygiene rules, and care for catheters [V52].

### 3.4. Financial Support Dimension

I can provide all kinds of assistance to individuals facing financial difficulties. I believe it’s important to buy them pajamas, nightgowns, and their favorite foods and fruits [V19].

Based on my observations, they often have significant financial needs [such as toilet paper, diapers, and paper towels]. I would like to cover these as much as my budget allows [V22].

I can provide financial assistance to patients in need. I can help with their shopping [V23].

I would buy small gifts for them [V49].

### 3.5. Spiritual Support Dimension

I would like to volunteer to support the fulfillment of patients’ psychosocial and spiritual needs [V51].

I would like to communicate with patients who have not yet lost consciousness and provide them with spiritual support. At the same time, I would support the patients’ relatives by talking about life and death as much as I can, giving them encouragement, and reflecting positively on the patient [V58].

### 3.6. Pediatric Palliative Care

I would like to tell stories to children using puppets because it is entertaining and motivating [V51, V56].

I would help children [V13].

I would play their favorite games with small children, read their favorite books, help with their lessons, and do arts and crafts with them. I would also buy gifts they would like and make treats such as cakes, cookies, candies, pastries, and börek [savory pastries] to bring to them [V28].

If our patient is a child, I would engage them in various games and entertainment activities to ensure they have a good time. If they have the opportunity to go outside, I would take them on outings, ensure they have an enjoyable experience, and assist in lightening the load for their parents [V44].

Since I understand and love children’s world very well, I would try to address any gaps in their education if they are unable to attend school. I could engage in activities like drawing, paper covering, puzzles, and paper cutting with them. I would fulfill all the needs that a mother could provide [V53].

With the necessary training provided to me, I can offer spiritual and psychological support to hospitalized children, read books to them, and perform massage therapy [V65].

## 4. Discussion

The rising demand for health and social care is driven by rapid population aging, social exchange, and care workforce shrinkage, which requires innovative initiatives to harness the time and energy of older adults [13]. Many older people in their 60s and 70s remain capable of contributing to society. Productive engagement encompasses unpaid and paid activities that generate goods and services for society, such as volunteering. Late-life volunteering has gained increasing attention in health promotion, as volunteering contributes to better health, the prevention of depression, and an improved quality of life among older adults. Promoting volunteering can also facilitate mutual help and strengthen the community’s caring capacity, potentially reducing the reliance on care professionals [14,15,16].

The average age of the Ege U3A students participating in the study was 67.47 (60–89) years. The approach of the older people towards voluntary services at U3A, where they actively engage in self-development and healthy aging, showed a significant change before and after the palliative care training we provided. Many older individuals who experience restrictions in their social lives frequently face issues such as social isolation, depression, and physical limitations. These health problems can partially reduce their potential for volunteering. With the training we provided, older individuals whose awareness of volunteering was strengthened and supported were encouraged to volunteer in specialized areas such as palliative care, despite their physical limitations. Their strength and motivation enabled them to contribute in meaningful ways. In a quantitative study conducted in Italy with a single-group pre-test/post-test design involving 19 older participants, it was reported that, after one year, there was an increase in the physical capacities, walking ability, and social participation of the participants [17]. Jenkinson and colleagues (2013) found in their review of forty experimental studies that older individuals who volunteer in their communities experience an improved quality of life and a reduction in depressive feelings and thoughts [18]. In our study, the U3A students expressed that social participation made them feel good, both psychologically and physiologically, and that helping others motivated them. The life experiences and instincts of U3A students to share their experiences in palliative care demonstrate that they can make the most significant contributions in the social support dimension.

Despite the increased participation rates of older adults in volunteer activities in developed countries in recent years, the advancements and research in this field remain insufficient [19,20]. In Türkiye, when examining volunteer activities, it was observed that sports organizations, religious groups, and social service charities dominate in terms of financial support, while rights-based organizations and support groups/NGOs make up a very small percentage. In Türkiye, volunteer activities often include charitable assistance to neighbors, families, and the poor; food aid during religious holidays and other special occasions; scholarships and educational support provided by foundations; assistance from organized civil society organizations; practices such as giving alms [zekat]. As a result, ongoing volunteer services often rely on financial support and are limited to a single type of activity [21]. Participation in sustained volunteer services is quite limited. The World Giving Index, published annually by the Charities Aid Foundation, periodically evaluates countries’ levels of volunteering by considering metrics such as making monetary donations to charities, spending time volunteering with organizations, and helping those in need or strangers. According to the latest report from 2022, which includes Türkiye, the Charities Aid Foundation’s World Giving Index ranked Türkiye 104th globally, with a participation rate of 11% in volunteer activities, including spending time or money on these activities [22]. This indicates that Türkiye is ranked quite low globally in terms of volunteer activities and is at an insufficient level [23].

For Turkish people, policymakers can encourage public awareness by preparing public service announcements, posters on public transportation, and short videos in subways, hospitals, and schools for recruiting volunteers. The need for volunteers increases as each day passes. Practices such as hiring people who are in an organization voluntarily could also be implemented, as is the case in European countries.

Older individuals have indicated that, when they are unable to provide physical assistance, they can offer financial support within their budget, prepare and deliver meals, and read books to others. The 2013 report “Volunteering and Older Adults” from Canada emphasizes similar support provided by volunteer seniors and highlights the importance of this support for both the country and the older person [24]. In our study, the older participants thoroughly grasped the need for social and psychological support for palliative care patients, as conveyed through the training content. In their written statements, they expressed a desire to assist with patients’ shopping, handle bureaucratic tasks [such as banking and paying bills], and provide spiritual support and motivation by either accompanying patients in palliative care services or taking them out of the service for a day. In Denmark, which implements volunteer services at the highest standards globally [25], it is noted that volunteers involved in strengthening social support networks not only provide support to the older person but also create motivation for themselves. Similar results have been highlighted in other European countries as well [26,27,28].

Our study highlights that older individuals, in particular, show a notable interest in volunteering in the field of pediatric palliative care. Older individuals, having reached a state of spiritual fulfillment and experiencing an increased frequency of loss within their social circles, are navigating the grieving process and grief psychology in a more meaningful way. In Korea, it has been highlighted that older volunteers demonstrate better adaptation to the grieving process and are more adept at managing end-of-life symptoms compared to university students working as pediatric palliative care volunteers [29,30]. Utilizing this potential volunteer strength of older people in pediatric palliative care is highly valuable.

In Türkiye, there is no infrastructure where individuals wishing to volunteer in palliative care can register or receive training to join this system. The services provided are conducted within closed structures of associations, foundations, and non-governmental organizations. In this area, it is necessary to create a database moving from general to specific and to begin by adapting a method suitable for Türkiye’s cultural structure from European countries that can successfully maintain this practice. In Turkish culture, although the extended family structure, which traditionally took care of older people at home with its own resources, has evolved into a nuclear family structure, there is still an understanding that families will try to take care of older people with their own means. Alongside the need to preserve this cultural structure, an urgent social support network should be developed for older people who are increasingly forced to live alone, and this network should be primarily provided by the state.

## 5. Conclusions

In our research, it was observed that older individuals with a high profile of volunteering are willing to work within their physical limitations and resources, and they recognize the need to support patients in this field. When older individuals receive conscious and systematic educational support based on their life experiences, it becomes more feasible and effective to harness this existing potential for volunteering. No previous pilot studies on older volunteers in palliative care have been conducted in Türkiye, according to our research, making this study a pioneering contribution to the volunteer database in this field. The next step of our research will be to train individuals who will volunteer in these areas and ensure their active participation in palliative care services. This will serve as an effective starting point and a motivating application for the development of a volunteer pool in Türkiye.

## 6. Limitations

Our study was conducted on a limited population of individuals aged 60 and above who had adopted a lifelong learning goal. A more extensive profile analysis on a broader population was not possible, which is a limitation of our research. A group with a high potential to demonstrate a willingness to volunteer was included in this research; therefore, the lack of inclusion of people with physical disabilities or those who see volunteering as an emotional burden is one of the limitations of this research. One of the limitations is that the research results were evaluated immediately after the training, and a similar volunteer motivation could not be evaluated in the long term.

## Data Availability

Data are contained within the article.

## Abbreviations

U3A: Ege University of the Third Age; WHO: The World Health Organization.
